# Cross-cultural adaptation of evidence-based practice measure among Hong Kong healthcare providers

**DOI:** 10.1371/journal.pone.0351754

**Published:** 2026-06-26

**Authors:** Fadi M. Al Zoubi, André Bussières, Jason P. Y. Cheung, Eric Chun-Pu Chu, Hammoda Abu-Odah, Arnold Y. L. Wong, Aliki Thomas

**Affiliations:** 1 Department of Rehabilitation Sciences, The Hong Kong Polytechnic University, Hong Kong SAR, China; 2 Research Institute for Smart Ageing, The Hong Kong Polytechnic University, Hong Kong SAR, China; 3 Faculty of Medicine and Health Sciences, School of Physical and Occupational Therapy, McGill University, Montreal, Quebec, Canada; 4 Département Chiropratique, Université du Québec à Trois-Rivières, Trois-Rivières, Quebec, Canada; 5 Department of Orthopaedics and Traumatology, The University of Hong Kong, Hong Kong SAR, China; 6 New York Chiropractic and Physiotherapy Centre, Hong Kong SAR, China; 7 Li Ka Shing Faculty of Medicine, School of Nursing, The University of Hong Kong, Pokfulam, Hong Kong SAR, China; 8 Centre for Interdisciplinary Research in Rehabilitation of Greater Montreal, Montreal, Quebec, Canada; 9 Faculty of Medicine and Health Sciences, Institute of Health Sciences Education, McGill University, Montreal, Quebec, Canada; University of Hafr Al-Batin, SAUDI ARABIA

## Abstract

**Objective:**

This study aimed to adapt and validate an Evidence-Based Practice (EBP) tool for healthcare providers in Hong Kong, addressing the need for reliable and culturally relevant tools.

**Methods:**

The original EBP measure, developed for recent Canadian physiotherapy and occupational therapy graduates, was systematically adapted for use by healthcare providers in Hong Kong following established cross-cultural adaptation guidelines. The adaptation process consisted of two phases: Phase I involved forward and backward translation, expert panel review, and cognitive debriefing interviews with a purposive sample of 36 healthcare providers to ensure linguistic and cultural relevance. In Phase II, the finalized instrument was administered to a convenience sample of 248 registered healthcare providers in Hong Kong. The structure of the adapted instrument remained consistent with the original, comprising two models encompassing six constructs: (1) a formative model (use of EBP and EBP activities) and (2) a reflective model (knowledge, self-efficacy, attitudes, and resources). Construct validity was assessed using the Rasch model, internal consistency reliability was evaluated with the Person Separation Index (PSI), and differential item functioning (DIF) was examined.

**Results:**

The formative model only required linguistic modifications. The Rasch model was applied to the reflective component. For knowledge, 45% (5/11) items fit the Rasch model with a chi-square fit statistic (χ² = 17.90, p = 0.268; PSI = 0.84). For self-efficacy, 89% (8/9) items fit the Rasch model with χ² = 26.48, p = 0.33; PSI = 0.93. The attitudes construct was divided into positively and negatively worded subscales due to multidimensionality. The resources construct showed a good fit with χ² = 26.60, p = 0.49; PSI = 0.84. DIF was not observed in the final measures.

**Conclusions:**

The adapted EBP measure demonstrated evidence of construct validity and internal consistency reliability among healthcare providers in Hong Kong. However, further research is needed to assess additional aspects of validity, such as test–retest reliability and responsiveness, as well as to evaluate its applicability in broader healthcare settings.

## Introduction

Evidence-Based Practice (EBP) is a systematic approach to healthcare that integrates the best available research evidence with clinical expertise and patient preferences to inform care [1]. It involves formulating precise questions, searching for relevant evidence, critically appraising that evidence, applying it in the clinical context, and evaluating outcomes to improve future practice [2]. In this study, the term “measure” refers to a validated questionnaire or psychometric tool designed to assess various EBP-related competencies and perceptions among healthcare providers, rather than an audit or compliance indicator.

EBP is a key competency for healthcare professions [3,4] such as nursing, physical therapy, and occupational therapy and is integrated into entry-level curricula. While the integration of EBP is widely recognized as a core competency in healthcare professions internationally [4–8], with explicit expectations articulated in the professional codes of conduct and practice of international federations, confederations, and regulatory councils, the situation in Hong Kong is less clear. To our knowledge, current professional codes of conduct for healthcare providers in Hong Kong do not explicitly require the implementation of EBP. This absence of a formal mandate may influence healthcare providers’ engagement with EBP and their responses to EBP-related measures. It also underscores the importance of culturally and contextually relevant assessment tools, as local professional norms and regulatory frameworks can significantly shape both attitudes toward and the adoption of EBP.

Research on how best to teach and evaluate EBP remains evolving [9–12]. Reviews of EBP educational interventions have shown that critical appraisal and the application of evidence in practice are often underemphasized [9–12]. In parallel, an umbrella review identified 204 distinct EBP measures, but many lacked thorough psychometric validation and failed to encompass the entire EBP process [13]. Existing tools tend to focus on selected domains rather than providing a comprehensive assessment of EBP [14], including both individual competencies and organizational supports [9,10,12]. These limitations highlight the need for a more robust and contextually relevant EBP measure.

### Study context

To address these measurement gaps, we previously developed a validated 68-item EBP measure in English and French for recent Canadian physiotherapy and occupational therapy graduates [15]. The measure includes both formative and reflective model constructs and captures individual as well as organizational dimensions of EBP. Although it has proven reliable and valid in its original context, cross-cultural adaptation is necessary before use in other cultural and professional settings [16].

We recently conducted a cross-cultural adaptation of the measure for use with Hong Kong physiotherapy students, demonstrating its reliability and validity in capturing both individual and organizational dimensions of EBP [17]. However, healthcare providers often differ significantly from students or recent graduates in terms of clinical exposure and professional responsibilities. Therefore, there is a need to expand the validation of the EBP measure to a broader group of healthcare providers in Hong Kong, including physiotherapists, occupational therapists, medical doctors, chiropractors, and nurses. This broader approach enables future comparisons of EBP competencies across different healthcare professions and better reflects the multidisciplinary nature of clinical practice in Hong Kong. Accordingly, the current study aimed to cross-culturally adapt the EBP measure for Hong Kong healthcare providers and rigorously evaluate its psychometric properties, ensuring its applicability across a broader spectrum of professional experience and practice contexts.

## Materials and methods

This study adhered to the STrengthening the Reporting of OBservational studies in Epidemiology (STROBE) checklist to ensure methodological rigor and transparency in reporting [18]. Ethical approval was obtained from the Institutional Review Board of The Hong Kong Polytechnic University (reference number: HSEARS20201208001). All participants gave electronic informed consent.

### Design

This study used a cross-sectional validation design to adapt and evaluate the psychometric properties of the Canadian EBP measure for use among Hong Kong healthcare providers. The research followed a two-step procedure. The first step focused on the cross-cultural validation of the EBP measure. This involved first translating the measure into Traditional Chinese, followed by conducting focus groups and pilot testing to assess and refine its linguistic clarity and cultural appropriateness for healthcare providers in Hong Kong. In the pilot-testing and focus meetings, we recruited medical doctors, registered nurses, physiotherapists, and chiropractors. The second step entailed the cross-sectional administration of an e-survey to a sample of practicing Hong Kong providers to assess the psychometric properties of the translated EBP measure, with a particular emphasis on evaluating structural validity and internal consistency reliability. Data collection was conducted in two phases. Phase I (linguistic and cultural validation) took place from March 29, 2021, to June 28, 2021, while Phase II (psychometric validation) was conducted from June 29, 2021, to March 28, 2022.

### EBP measure

We adapted the EBP measure originally developed in English and validated among Canadian occupational therapy and physiotherapy graduates [15]. Since Hong Kong healthcare providers are trained and practice in English, the Canadian version was considered suitable for adaptation. However, it is important to note that while English is widely used in Hong Kong, there are differences in vocabulary, usage, and cultural context compared to Canadian English. Therefore, the measure was further reviewed and adapted to ensure appropriateness for Hong Kong English and local clinical practice. The measure consists of six constructs—use of EBP, EBP activities, knowledge, self-efficacy, attitudes, and resources—encompassing a total of 68 items. Each construct is described in Table 1.

**Table 1:** Description of the EBP measure constructs.

Constructs	Number of items	Response options
*Use of EBP*	9	Assesses the actual application of EBP, reflecting the five steps of the EBP process [2] over the past six months. Responses are recorded on a 5-point scale: *‘Never,’ ‘1 to 2 times,’ ‘Almost every month,’ ‘2 to 10 times a month,’ and ‘More than 10 times a month.’*
*EBP Activities*	7	Evaluates the integration of research findings into practice, such as formally or informally sharing recent research with colleagues or patients in the workplace or learning environment. Responses are captured on a 5-point scale ranging from *‘never’* to *‘daily,’* with total scores ranging from 0 to 140. Higher scores indicate greater engagement in EBP activities.
*Knowledge about EBP*	11	Assesses participants’ understanding of fundamental EBP concepts, including research terminology, statistical methods, and methodological principles. Responses are recorded on a 5-point scale: *‘Never heard the term,’ ‘Have heard it but do not understand,’ ‘Have some understanding,’ ‘Understand quite well,’* and *‘Understand and could explain to other.’* Higher scores reflect greater EBP knowledge.
*Self-efficacy*	9	Measures participants’ confidence in their ability to implement the five steps of EBP in clinical practice. Responses are recorded on an 11-point scale ranging from *‘0% (no confidence)’* to *‘100% (complete confidence),’* with higher percentages indicating greater self-efficacy.
*Attitudes towards EBP*	17	evaluates healthcare providers’ perceptions of EBP. All items use a 5-point Likert scale, ranging from *‘strongly disagree’* to *‘strongly agree.’*
*EBP resources*	15	Assesses the availability of resources that support the implementation of EBP in the workplace. Responses are recorded on a 5-point Likert scale, ranging from *‘strongly disagree’* to *‘strongly agree.’*

### Phase I: Linguistic and cultural validation

To ensure cultural relevance and linguistic clarity, the measure was adapted from North American English to Hong Kong English, following guidelines for cross-cultural adaptation [16]. We utilized a six-step adaptation process:

i. *Forward translation*: Three local translators, fluent in Hong Kong English, independently reviewed the original North American English EBP measure. Their task was to identify and adjust terms or phrases to enhance comprehension among local healthcare providers. One translator had a background in physiotherapy with 15 years of experience and familiarity with the research topic and EBP questionnaires, which provided subject-matter insight into the conceptual meaning of the items. The other two translators did not have medical backgrounds, and they have degrees in English translation with 2 and 10 years of experience and were chosen to provide a lay perspective and to suggest alternative phrasing to improve clarity and understanding. All translators were bilingual in English and traditional Chinese and were selected based on language proficiency and relevant experience. Each translator first reviewed the measure independently, after which they discussed their suggestions as a group.
ii. *Translation synthesis*: After the independent review, the three translators convened for a synthesis meeting. During this meeting, each translator presented their suggested translations and the rationale behind their choices. The group systematically compared each item, discussed any differences in wording or interpretation, and collaboratively resolved discrepancies through discussion and consensus. If disagreements arose, the group considered the clarity, cultural appropriateness, and intended meaning of each item in order to reach a decision. A physiotherapy professor (AW) acted as the synthesis recorder and facilitator, guiding the discussion, ensuring that all viewpoints were considered, and mediating any unresolved disagreements. The outcome of this process was a single, harmonized draft version of the EBP measure that reflected both professional and lay perspectives.
iii. *Back translation*: This step was deemed unnecessary, as the adaptation involved transitioning between two forms of English rather than distinct languages.
iv. *Expert committee review*: An expert committee, comprising the forward translators, the synthesis recorder (AW), two North American English-speaking EBP experts (AT, AB), and an experienced researcher in outcome measures and EBP (FAZ), reviewed the draft. During two separate meetings, the committee evaluated the measure for semantic and idiomatic accuracy, ensuring conceptual and experiential equivalence. The revised preliminary version of the measure was approved by all members of the expert committee.
v. *Test of the pre-final version*: This pre-final English Hong Kong version was tested with a purposive sample of healthcare providers, including physiotherapists, occupational therapists, medical doctors, chiropractors, and nurses. In line with established recommendations for cross-cultural validation of measurement instruments [16], 30–40 participants were invited to review the measure for clarity, relevance, and cultural appropriateness. The sample was selected to ensure representation across a range of healthcare professions that would use the EBP measure in practice. Selection criteria included being an adult (18 years or older), having graduated from a healthcare program, and being registered with the local licensing board. Participants were contacted via email or professional networks. Email contact details were obtained through the researchers’ existing professional networks and contacts within the local healthcare community. Upon providing informed consent, participants were first asked to independently complete the pre-final version of the EBP measure. Following completion, a trained research assistant with 7 years of experience in quantitative and qualitative research as a psychologist conducted a semi-structured cognitive debriefing interview with each participant, either in person or via videoconference. Before data collection, the research assistant received study-specific training from the research team on informed consent procedures, administration of the semi-structured interview guide, and techniques for conducting cognitive debriefing interviews consistently and neutrally. During the interview, participants were asked to rate each item’s relevance to EBP on a 5-point Likert scale (ranging from ‘not relevant at all’ to ‘extremely relevant’) and to assess the comprehensibility of each item on a 5-point scale (from ‘very difficult to understand’ to ‘very easy to understand’). Participants were also encouraged to discuss any items they found unclear, confusing, or culturally inappropriate and to suggest alternative wording where necessary. This process followed established recommendations for cross-cultural validation [16], ensuring both quantitative and qualitative feedback was systematically collected to inform further refinement of the measure. We recognize that participants’ judgments of item relevance during cognitive debriefing may have been influenced by their own level of EBP knowledge and confidence. To mitigate this, we recruited a diverse sample and instructed participants to consider the broader relevance of each item to the corresponding EBP construct in their profession. The expert panel also reviewed all feedback to ensure that item retention was guided by both participant input and the conceptual framework of EBP. Interviews were audio-recorded, transcribed verbatim, and systematically analyzed using deductive thematic analysis [19] to identify issues related to the measure’s relevance, comprehensiveness, and cultural appropriateness. Key themes and participant suggestions were extracted and summarized.
vi. *Final version and appraisal of the adaptation*: The summarized findings from the cognitive debriefing interviews —including both quantitative ratings and qualitative themes— were presented to the expert panel for review. The panel discussed the results and made recommendations for final adjustments to the measure. Based on these recommendations, necessary revisions were incorporated. The finalized Hong Kong English version was then sent to the original North American developers for review and approval, ensuring its alignment with the intended purpose and conceptual framework of the EBP measure.

### Phase II: Psychometric validation

The psychometric properties of the EBP measure were rigorously evaluated in accordance with established guidelines [20–22]. Structural validity was specifically assessed using Rasch analysis, a robust statistical method for analyzing ordinal data and ensuring the measure’s unidimensionality and reliability [15].

#### Participants.

A convenience sampling approach was used to recruit a diverse group of healthcare providers in Hong Kong. Eligible participants were registered healthcare professionals—including physiotherapists, occupational therapists, medical doctors, chiropractors, nurses, and other allied health providers—who had attended institutions where English was the primary language of instruction. Recruitment was conducted through professional bodies and associations, such as the Hong Kong Physiotherapy Association and Chiropractic Doctors’ Association of Hong Kong, along with postings in academic departments (e.g., Department of Rehabilitation Sciences at The Hong Kong Polytechnic University and Department of Orthopaedics and Traumatology, The University of Hong Kong) and clinics. Invitations were distributed via email lists, newsletters, and online platforms managed by these organizations. Inclusion criteria were (1) currently registered and practicing in Hong Kong during the study period (2021–2022) and (2) able to read and understand English. There were no restrictions regarding years of experience, clinical specialty, or workplace setting. Following methodological guidelines, a minimum sample size of 200 participants was targeted to ensure sufficient statistical power for Rasch analysis to yield stable and reliable estimates of the measure’s psychometric properties [23–25].

#### Recruitment and data collection.

Participants were recruited through three main methods: the graduate member lists of healthcare programs; bulk email invitations from the Hong Kong Physiotherapy Association and the Chiropractic Doctors’ Association of Hong Kong; along with postings in academic departments (e.g., Department of Rehabilitation Sciences at The Hong Kong Polytechnic University and Department of Orthopaedics and Traumatology, The University of Hong Kong) and clinics. All three methods outlined the study’s objectives and the estimated 10-minute survey completion time. The survey was created using Qualtrics software (Qualtrics Survey2020, Utah, USA; https://www.qualtrics.com/). It began with two pages for the consent form and demographic data, followed by six pages focused on evaluating each construct within the EBP measure. Participation in both Phase I and Phase II was voluntary, and no financial or non-financial compensation was provided.

#### Data analysis.

Descriptive statistics were used to summarize the data. Continuous variables were reported as means and standard deviations (SD), while categorical variables were presented as frequencies and percentages. All statistical analyses were conducted using the Statistical Package for the Social Sciences (SPSS v.26) [26]. Additionally, Rasch analyses were performed using the Rasch Unidimensional Measurement Model (RUMM) software (version 2030) [27] to evaluate the structural validity and reliability of the EBP measure.

#### Rasch analysis.

The reliability and validity of the EBP measure were evaluated using Rasch analysis [15]. Rasch analysis is a robust psychometric technique within the framework of item response theory, designed to transform ordinal data into interval-level data, enabling more precise measurement [28]. This model organizes items hierarchically based on their difficulty, aligning them with the ability range of respondents. Specifically, individuals with higher abilities (e.g., those who practice EBP more frequently) are more likely to select advanced response options, while those with lower abilities tend to choose less advanced options. This hierarchy is visually represented through an item map, which illustrates the distribution of items along a continuum from least to most difficult [28]. Rasch analysis assumes unidimensionality; therefore, differential item functioning (DIF) evaluations are paramount to ensure that items operate consistently across different subgroups. Applying these psychometric properties is essential to verify the reliability, validity, and scoring methods of any instrument [29].

The Masters’ partial credit Rasch polytomous model was employed, as it is well-suited for analyzing ordinal response options [30]. As outlined in our initial study [15], two constructs—EBP usage and EBP activities—are formative in nature and thus do not require Rasch analysis. Conversely, the four reflective constructs—attitudes, self-efficacy, knowledge, and resources—were subjected to Rasch analysis to assess their psychometric properties. Table 2 details the Rasch analysis steps we applied to its assumptions.

**Table 2:** Procedural framework for Rasch model data fitting.

Statistical step	Description
Item response thresholds	Each item within the four EBP constructs was polytomous, featuring at least five response categories. The thresholds between categories represent critical points on the latent variable where the probability of selecting one category over another is equal [31,32]. These thresholds must logically order to reflect increasing levels of the latent variable, a principle known as monotonicity. This is crucial for the Rasch model, ensuring that responses align with the measured construct. To confirm monotonicity, we analyzed item threshold parameters, maps, and category probability curves. If thresholds were disordered, suggesting misalignment with the variable’s progression, we merged adjacent categories. This sometimes led to a binary item format, simplifying responses while maintaining measurement integrity, ensuring that the items accurately mirrored the latent variable in accordance with Rasch model expectations.
Overall, person and item fit to the Rasch model	The overall model fit was assessed using summary fit residual statistics. Items were deemed to fit the Rasch model if the chi-square fit statistic (χ²) and F-statistic were non-significant, with a p-value > 0.05 after applying a Bonferroni adjustment for multiple comparisons [31,32]. To evaluate how well the observed responses for each item and person aligned with the predictions of the Rasch model, we analyzed standardized fit residuals. Residual values falling within the range of ±2.5 indicated a good fit to the model, suggesting that the responses were consistent with the expectations of the Rasch framework [31,32]. However, residuals exceeding +2.5 may indicate potential item misfit and multidimensionality or noise in the scale, whereas residuals below −2.5 may indicate redundancy [33,34].
Unidimensionality	Unidimensionality—a core assumption of the Rasch model—requires that all items within a construct measure the same underlying latent variable. We conducted a principal component analysis (PCA) of the residuals, which identified two subsets of items with opposing loadings, which were then compared using independent t-tests [31,32]. For a construct to be considered unidimensional, fewer than 5% of these t-tests needed to yield significant results, with t-values falling outside the range of ±1.96 [35].
Structural validity	Structural validity was evaluated by examining the distribution of items along a hierarchical linear continuum, ranging from the easiest to the most difficult. This analysis involved both statistical and graphical inspections of item placements. Ideally, the locations of items and individuals on this continuum should be centered at 0, with a standard deviation (SD) of 1, reflecting a well-calibrated scale. During this process, we identified and removed redundant items—those occupying similar positions on the continuum—to ensure each item uniquely contributed to measuring the latent variable. We also inspected for gaps within the ± 4 logit range, identifying potential areas where the measure may not fully capture the underlying construct.
Local item dependence	Local item dependence occurs when responses to one item are influenced by another within the same construct, undermining the independence assumption crucial for robust measurement [37]. To detect such dependencies, we examined the residual correlation matrix, flagging item pairs with correlations over 0.3 as potential indicators of local item dependence. To address this issue, we used two strategies: merging response options of dependent items into a “super item” for a more robust measure, or retaining the item with the most precise phrasing while removing the dependent counterpart [37].
Differential item functioning (DIF)	DIF, also referred to as item bias, occurs when different subgroups within a sample exhibit varying levels of ability on the same item, despite having comparable overall proficiency. To evaluate DIF across the EBP constructs, we examined personal factors such as profession (medical doctors, physiotherapists (PTs), occupational therapists (OTs), nurses, and chiropractors (DCs)), clinical experience (<5 years, 5–10 years, 10–20 years, and >20 years), sex (male and female), and level of education (diploma, Bachelor of Science (BSc), Master of Science (MSc), and Doctor of Philosophy (PhD)). DIF was identified through a significant F-test derived from a two-way analysis of variance (ANOVA) and further visualized using item characteristic curve (ICC) plots for each personal factor. The sample size significantly affects the F-test’s significance. Samples exceeding 500 may increase the likelihood of detecting significant DIF, whereas smaller samples under 200 might not have sufficient power to identify it. Therefore, a sample size between 200 and 500 ensures a balance of sensitivity and reliability [23–25]. When DIF is detected, items are either adapted for subgroup-specific versions or removed to reduce bias and ensure fair measurement across all subgroups.
Internal consistency reliability	The internal consistency reliability of the constructs—knowledge, self-efficacy, attitudes, and resources—was evaluated using the Person Separation Index (PSI) and Cronbach’s alpha (α) [33,34]. Both PSI and Cronbach’s α values of ≥0.7 were considered indicative of strong internal consistency, reflecting the reliability of the constructs in measuring the intended latent variables [33,34].

## Results

### Phase I: Linguistic and cultural validation

The translation and cultural adaptation of the EBP measure were systematically carried out through steps I to IV of phase I. During this process, forward translators proposed several modifications to simplify the language, which were subsequently reviewed and approved by the expert committee. In phase V, cognitive debriefing was conducted with 36 participants to assess the relevance and comprehensibility of the adapted items. The demographic characteristics of the cognitive debriefing sample are presented in Table 3. Feedback from the interviews indicated that the EBP items were relevant to their respective constructs, easily understandable, and required an average completion time of 15 minutes. A detailed summary of the changes made to the EBP constructs’ items, along with additional insights from the cognitive debriefing process, is further detailed in S1 File.

**Table 3:** Summary of the participants main characteristics.

Participant Characteristics	Cognitive Debriefing (n = 36)	Psychometric testing (n = 248)
Age: (Mean (SD)	36.5 (12.0)	36.3 (10.8)
Sex: n (%)		
Male	15 (41.7%)	127 (51.2%)
Female	21 (58.3%)	121 (48.8%)
Healthcare providers: n (%)		
Medical doctors	8 (22.2%)	32 (12.9%)
Registered nurse	1 (2.8%)	16 (6.5%)
Physiotherapist	12 (33.3%)	119 (48.0%)
Occupational therapist	0 (0.0%)	45 (18.1%)
Chiropractor	15 (41.7%)	36 (14.5%)
Year of practice (years): (Mean (SD)	11.8 (11.1)	12.3 (10.3)
Year in current setting: (Mean (SD)	6.6 (8.0)	7.5 (8.1)
Education level: n (%)		
Diploma	0 (0%)	7 (2.8%)
Bachelor degree	15 (41.7%)	87 (35.1%)
MSc degree	16 (44.4%)	137 (55.2%)
PhD degree	5 (13.9%)	17 (6.9%)
Work setting: n (%)		
*Single setting*	30 (83.3%)	218 (87.9%)
Hospital Authority	1 (2.8%)	78 (35.8%)
Private Hospital	7 (19.4%)	8 (3.7%)
NGO	0 (0%)	55 (25.2%)
Primary health care	1 (2.8%)	3 (1.4%)
Special school	0 (0%)	4 (1.8%)
Home visiting agency	0 (0%)	2 (0.9%)
Old age home	0 (0%)	3 (1.4%)
Private practice	20 (55.6%)	62 (28.4%)
School-affiliated setting	0 (0%)	3 (1.4%)
*Multiple setting*	6 (16.7%)	30 (12.1%)
Work hour per week: n (%)		
<8 hours	0 (0%)	10 (4.0%)
8-14 hours	3 (8.3%)	6 (2.4%)
15-21 hours	1 (2.8%)	24 (9.7%)
22-28 hours	1 (2.8%)	42 (16.9%)
29-35 hours	0 (0%)	81 (32.7%)
36-42 hours	12 (33.3%)	61 (24.6%)
43-49 hours	7 (19.4%)	12 (4.8%)
> 50 hours	12 (33.3%)	12 (4.8%)
Patient per day: (Mean (SD)	15.2 (11.5)	23.2 (19.0)
No. of colleagues: (Mean (SD)	20.4 (29.5)	21.5 (35.3)
Interdisciplinary team: n (%)		
Yes	29 (80.6%)	200 (80.6%)
No	7 (19.4%)	48 (19.4%)
Professional development: n (%)		
Yes	35 (97.2%)	241 (97.2%)
No	1 (2.8%)	7 (2.8%)
University affiliated: n (%)		
Yes	4 (11.1%)	63 (25.4%)
No	22 (31.1%)	147 (59.3%)
Don’t know	0 (0%)	38 (15.3%)

SD: Standard Deviation; n: Number; MSc: Master of Science; PhD: Doctor of Philosophy; NGO: Non-Governmental Organization.

Weekly work-hour categories were created in approximately 7-hour increments for descriptive purposes to reflect increasing workload ranges.

### Phase II: Psychometric validation

Of the 640 users who accessed the study link, 248 (119 PTs, 45 OTs, 36 DCs, 32 medical doctors, and 16 registered nurses) successfully completed the e-survey. Detailed demographic and professional characteristics of the participants are presented in Table 3.

#### Formative model (use of EBP and EBP activities).

For the *Use of EBP* construct, items remained unchanged during the linguistic and cultural adaptation phase, ensuring consistency with the original North American instrument. The scoring approach for this construct was retained from the original Canadian EBP measure [15], in which Use of EBP was conceptualized as a formative construct. Each of the 9 items includes 5 frequency-based response options referring to the past 6 months; however, for scoring purposes, responses are dichotomized and assigned a score of either 0 or 1. Specifically, selecting “Never” (indicating no use) is scored as 0, while selecting any of the other options (indicating one or more instances of use) is scored as 1. This total score reflects the number of EBP behaviors performed rather than the intensity or exact frequency of performance. This cutoff was adopted from the original instrument to indicate whether each core EBP behavior had been undertaken at least once during the reference period. Accordingly, the total score reflects the number of EBP behaviors performed, rather than their exact frequency or intensity. The total score is obtained by summing the scores for all 9 items, yielding a possible range from 0 to 9. A detailed breakdown of the scoring methodology is provided in Table 4.

**Table 4:** Results of analysis for the “Current self-reported use of EBP” measure.

*Instructions:* *In the past 6 months, how often have you? 5-point Scale*						
Item #	Description of Item:	Never	1 to 2 times	Almost every month	2 to 10 times a month	More than 10 times a month
1	Identified a gap in your knowledge related to a patient or client situation (e.g., history, assessment, treatment)?	0	1			
2	Formulated a question to guide a literature search based on a gap in your knowledge?	0	1			
3	Effectively conducted an online literature search to address the question?	0	1			
4	Critically appraised the strengths and weaknesses of research methods (e.g., appropriateness of study design, recruitment, data collection and analysis)?	0	1			
5	Critically appraised the measurement properties (e.g., reliability and validity, sensitivity and specificity) of standardized tests or assessment tools you are considering using in your practice?	0	1			
6	Interpreted study results with the use of statistical tests and procedures (e.g., t-tests, logistic regression?)	0	1			
7	Determined if evidence from the research literature applies to your patient’s/client’s situation?	0	1			
8	Determined on an appropriate course of action based on integrating the research evidence, clinical judgment and patient or client preferences?	0	1			
9	Continually evaluated the effect of your course of action on your patient’s/client’s outcomes?	0	1			

For the *EBP Activities* construct, the original scoring system was preserved, including the method for calculating the total score to maintain consistency with the source instrument [15]. Unlike the **Use of EBP** construct, these items were **not dichotomized**. Instead, response options were weighted to approximate the frequency with which each activity was performed over the previous month. Specifically, the categories were scored as follows: “Never” = 0, “Monthly or less” = 1, “Bi-weekly” = 2, “Weekly” = 4, and “Daily” = 20. These weights were designed to reflect the approximate number of occasions per month on which the activity occurred, with “Daily” corresponding to an estimated 20 working days in a 4-week month. As this construct is formative, the total score should be interpreted as a practical index of engagement in EBP-related activities rather than as a reflective scale with equally spaced psychometric intervals. During the pilot study (Phase I), participants agreed that the response options accurately reflected their frequency of engagement in the listed activities. The expert committee reviewed this feedback and endorsed it without any further adjustments. A summary of the results for the EBP Activities measure is provided in Table 5.

**Table 5:** Results of analysis for the “EBP activities” measure.

*Instructions:* *In the* *past month* *how often have you?: 5-point Scale*						
Item #	Description of Item	Never	Monthly or less	Bi-weekly	Weekly	Daily
10	Integrated research evidence with your expertise	0	1	2	4	20
11	Informally (e.g., hallway chatting) shared and discussed literature/research findings with colleagues at work	0	1	2	4	20
12	Formally (e.g., during team meetings) shared and discussed literature/research findings with colleagues at work	0	1	2	4	20
13	Shared and discussed literature/research findings with patients/clients	0	1	2	4	20
14	Read published research reports	0	1	2	4	20
15	Spent time to read research	0	1	2	4	20
16	Attended in-services/workshops/courses in your organization?	0	1	2	4	20

### Reflective constructs

#### Knowledge about EBP.

Table 6 presents the Rasch analysis results for the original 11 items assessing EBP knowledge. Initially, seven items exhibited disordered thresholds, prompting the rescoring of these items by merging the response categories *“Never heard the term”* and *“Have heard it but don’t understand”* (items 34, 35, 36, 39, 41, 42, and 44). Following this adjustment, six items were removed due to a misfit with the Rasch model. Three items (39, 40, and 42) were excluded based on fit residuals, while the remaining three (34, 41, and 44) were removed due to significant χ² and F-statistics.

**Table 6:** Rasch analysis results for the Rasch model for the “Knowledge about EBP” measure.

a. Fit of data to the Rasch model (n = 230).												
Analysis	N of items	Location, x (SD)		Fit residuals, x (SD)		χ² interaction		Reliability		Dimensionality	Local item independence	DIF
		Item	Person	Item	Person	χ^2^ (df)	*p*	PSI	Cronbach’s α	% t-tests >5%		
**A1**	11	0.0 (1.07)	1.52 (2.17)	−0.33 (1.69)	−0.73 (1.74)	101.91 (33)	0.000	0.92	0.94	11.76	34 + 35, 41 + 44	34, 40, and 44 by profession
**A2**	11	0.0 (0.62)	−0.43 (1.86)	0.74 (2.06)	−0.87 (1.74)	91.65 (33)	0.000	0.92	0.94	10.92	34 + 35	34, 40, and 44 by profession
**A3**	10	0.0 (0.29)	0.92 (2.13)	−0.54 (1.81)	−0.86 (1.81)	42.29 (30)	0.068	0.92	0.93	10.09	34 + 35	34, 40, and 44 by profession
**A4**	9	0.00 (0.23)	1.00 (2.22)	−0.72 (1.66)	−0.94 (1.82)	61.64 (27)	0.000	0.91	0.93	7.14	34 + 35	37 by profession
**A5**	8	0.00 (0.23)	0.97 (2.15)	−0.62 (1.61)	−0.88 (1.70)	54.66 (24)	0.000	0.90	0.92	7.56	34 + 35	37 by profession
**A6**	7	0 (0.24)	0.96 (2.09)	−0.36 (1.10)	−0.75 (1.52)	39.00 (21)	0.01	0.89	0.91	5.04	None	37 by profession
**A7**	6	0 (1.8)	0.90 (1.05)	−0.46 (0.99)	−0.86 (1.64)	25.09 (18)	0.12	0.87	0.89	5.02	None	37 by profession
**A8**	5	0 (0.21)	0.90 (2.02)	−0.59 (1.04)	−0.91 (1.65)	17.90 (15)	0.268	0.84	0.88	4.99	None	37 by profession (left)
***Ideal value***		*0 (1.0)*	*0 (1.0)*	*<1.4*	*<1.4*		*>0.05*	*>0.7*	*>0.7*	*< 5.0*	*<0.3 below average residual*	*None*
**b. Changes applied to the “Knowledge about EBP” items**												
**Item #**	**Description of Item**						**Never heard the term**		**Have heard it but don’t understand**	**Have some understanding**	**Understand quite well**	**Understand and could explain to others**
34	Reliability of outcome measures						**Deleted because of misfit**					
35	Validity of outcome measures						0		0	1	2	3
36	Sensitivity/Specificity of outcome measures						0		0	1	2	3
37	Meta-analysis						0		1	2	3	4
38	Confidence Interval						0		1	2	3	4
39	Systematic Review						**Deleted because of misfit**					
40	Number needed to treat						**Deleted because of misfit**					
41	Statistical significance						**Deleted because of misfit**					
42	Minimally important change (MIC)						**Deleted because of misfit**					
43	Treatment effect size						0		1	2	3	4
44	Randomized controlled trial (RCT)						**Deleted because of misfit**					

x: Mean; SD: Standard deviation; χ²: Chi-squared; df: Degrees of freedom; DIF: Differential item functioning; PSI: Person separation index; α: Alpha.

A1: Explore items.

A2: Rescore disordered thresholds of items 34, 35, 36, 39, 41, 42, and 44.

A3: Remove item 42 as it misfits the Rasch model (fit residual = 2.625, χ^2^ = 15.472, p-value = 0.0015, F-stat = 3.896, p-value for F-stat = 0.0097).

A4: Remove item 40 as it misfits the Rasch model (fit residual = 3.050, χ^2^ = 12.917, p-value = 0.0048, F-stat = 3.630, p-value for F-stat = 0.0137).

A5: Remove item 39 as it misfits the Rasch model (fit residual = −2.978, χ^2^ = 8.201, p-value = 0.0420, F-stat = 4.672, p-value for F-stat = 0.0034).

A6: Remove item 34 as it misfits the Rasch model (fit residual = −2.688, χ^2^ = 5.418, p-value = 0.1436, F-stat = 3.38, p-value for F-stat = 0.0299).

A7: Remove item 44 as it misfits the Rasch model (fit residual = −0.968, χ^2^ = 13.569, p-value = 0.0036, F-stat = 6.601, p-value for F-stat = 0.0003).

A8: Remove item 41 as it misfits the Rasch model (fit residual = −0.426, χ^2^ = 8.422, p-value = 0.0380, F-stat = 3.423, p-value for F-stat = 0.0181).

Local dependency was initially observed between items 34 and 35, as well as between items 41 and 44. However, these dependencies were resolved in the final analysis after the removal of misfitting items. DIF was identified for item 37 (*Meta-analysis*) by profession, but the item was retained due to its relevance and contribution to the construct.

**Fig 1:**
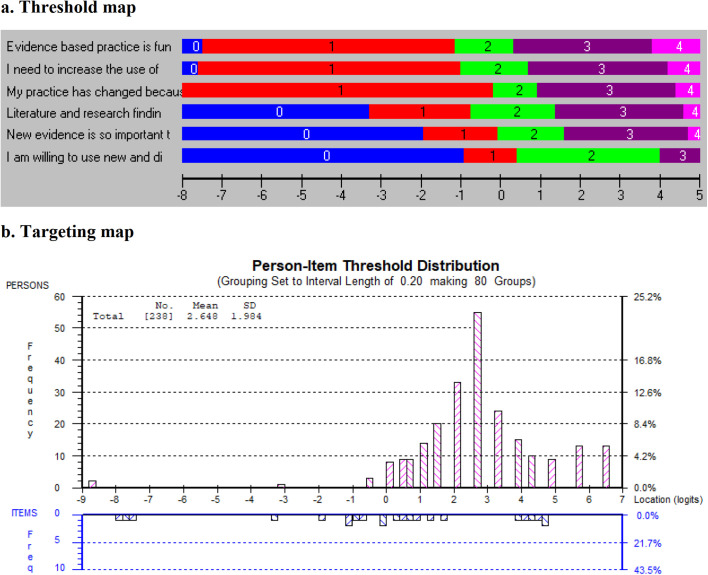
a. Threshold map and b. Targeting map of the “Positively-worded Attitudes towards EBP” construct.

The final 5-item measure demonstrated excellent fit to the Rasch model (χ² = 17.90, df = 15, p = 0.268), with no significant concerns regarding unidimensionality (% of significant t-tests = 4.99, below the 5% threshold). The threshold map, illustrated in Fig 1a, revealed a clear hierarchy in the difficulty of the knowledge items. Understanding *confidence intervals* was the least challenging, while grasping *treatment effect size* was the most difficult. Fig 1b shows that participants were reasonably well-targeted by the items, with a mean person location of 0.90 (expected 0) and an SD of 2.02 (expected 1). The measure also exhibited strong reliability, with a Person Separation Index (PSI) of 0.84 and a Cronbach’s α of 0.88, indicating high internal consistency.

#### Self-efficacy towards EBP.

The self-efficacy items related to EBP are outlined in Table 7. Initially, all items displayed disordered thresholds, requiring rescoring to ensure proper alignment with the Rasch model. For seven items (46, 47, 48, 50, 51, 52, and 53), the “90%” and “100%” response categories were merged. Item 45 was adjusted to two binary categories after rescoring. Item 49 had the “80%,” “90%,” and “100%” categories consolidated. Six items (46, 48, 50, 51, 52, and 53) involved combining the “0%,” “10%,” “20%,” and “30%” categories. Two items (47 and 49) merged the “0%,” “10%,” “20%,” “30%,” and “40%” categories. Following these modifications, item 53 was excluded due to its misfit with the Rasch model. The final 8-item measure showed no evidence of local item dependency or DIF, confirming its robustness.

**Table 7:** Rasch analysis results for the Rasch model for the “Self-efficacy towards EBP” measure.

a. Fit of data to the Rasch model (n = 235).												
Analysis	N of items	Location, mean (SD)		Fit residuals, mean (SD)		Chi-square interaction		Reliability		Dimensionality	Local item independence	DIF
		Item	Person	Item	Person	χ^2^ (df)	*p*	PSI	Cronbach’s α	% t-tests >5%		
**A1**	9	0 (0.37)	0.04 (2.19)	0.02 (2.37)	−1.06 (2.10)	43.23 (27)	0.025	0.96	0.97	14.04	48 + 49, and 52 + 53	None
**A2**	9	0 (0.64)	0.50 (2.49)	−0.21 (1.89)	−0.78 (1.66)	33.62 (27)	0.17	0.94	0.94	6.03	48 + 49, and 52 + 53	None
**A3**	8	0 (0.67)	0.49 (2.38)	−0.15 (1.26)	−0.69 (1.49)	26.48 (24)	0.33	0.93	0.95	4.87	None	None
***Ideal value***		*0 (1.0)*	*0 (1.0)*	*<1.4*	*<1.4*		*>0.05*	*>0.7*	*>0.7*	*< 5.0*	*<0.3 below average residual*	*None*
**b. Changes applied to the “Self-efficacy towards EBP” items.**												
***Instructions:*** *Please indicate how confident you are in your current level of ability by choosing the corresponding number on the following rating scale: 11-point Continuous Scale*												
**Item #**	**Description of Item**	**0% (No confidence)**	**10%**	**20%**	**30%**	**40%**	**50%**	**60%**	**70%**	**80%**	**90%**	**100% (Completely confident)**
45	Identify a gap in your knowledge related to a patient or client situation (e.g., history, assessment, treatment)?	0						1				
46	Formulate a question to guide a literature search based on a gap in your knowledge?	0				1	2	3	4	5	6	
47	Effectively conduct an online literature search to address the question?	0					1	2	3	4	5	
48	Critically appraise the strengths and weaknesses of study methods (e.g., appropriateness of study design, recruitment, data collection and analysis)?	0				1	2	3	4	5	6	
49	Critically appraise the measurement properties (e.g., reliability and validity, sensitivity and specificity) of standardized tests or assessment tools (that) you are considering using in your practice?	0					1	2	3	4		
50	Interpret study results obtained using statistical tests and procedures (e.g., t-tests, logistic regression?)	0				1	2	3	4	5	6	
51	Determine if evidence from the research literature applies to your patient’s/client’s situation?	0				1	2	3	4	5	6	
52	Decide on an appropriate course of action based on integrating the research evidence, clinical judgment and patient or client preferences?	0				1	2	3	4	5	6	
53	Continually evaluate the effect of your course of action on your patient’s/client’s outcomes?	**Deleted because of misfit**										

x: Mean; SD: Standard deviation; χ²: Chi-squared; df: Degrees of freedom; DIF: Differential item functioning; PSI: Person separation index; α: Alpha.

A1: Explore items and reverse item 65.

A2: Rescore disordered thresholds of all items.

A3: Remove item 53 as it misfits Rasch model (fit residual = −2.792, χ^2^ = 4.335, p-value = 0.0227, F-stat = 2.670, p-value for F-stat = 0.0486).

**Fig 2:**
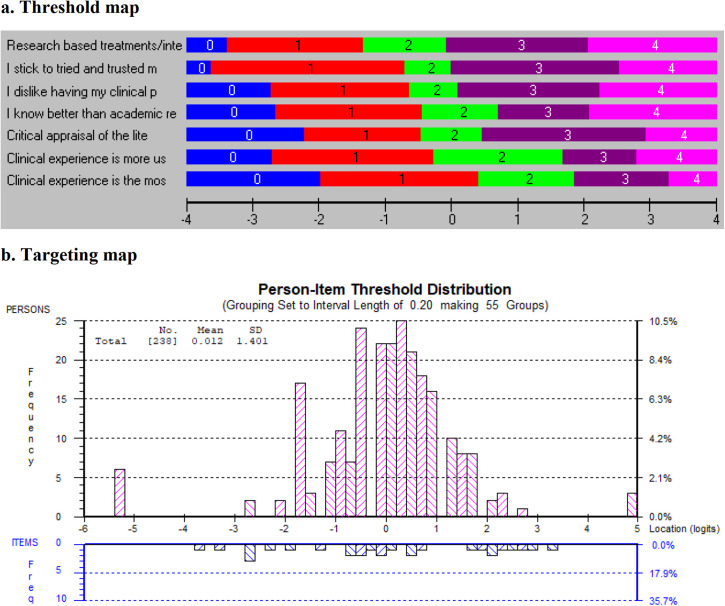
a. Threshold map and b. Targeting map of the “Negatively-worded Attitudes towards EBP” construct.

The model demonstrated excellent fit (χ² = 26.48, df = 24, p = 0.33), with no significant concerns regarding unidimensionality (% of significant t-tests = 4.87, below the 5% threshold). Fig 2a illustrates the progression of item thresholds, reflecting the hierarchical structure of the self-efficacy items. Fig 2b indicates that participants were well-targeted by the items, with a mean person location of 0.49 (expected 0) and an SD of 2.39 (expected 1). The final measure exhibited strong reliability, with a PSI of 0.93 and a Cronbach’s α of 0.95, underscoring its high internal consistency.

### Attitudes towards EBP

The attitudes construct was significantly multidimensional (% of significant t-tests = 25.85), comprising two distinct sets of items: positively worded (items 17–25) and negatively worded (items 26–33). Despite efforts to rescore and eliminate misfitting items, maintaining both sets within a single construct proved impractical. A bi-factor Rasch analysis confirmed their lack of unidimensionality, necessitating separate analyses for each set.

#### Positively worded attitudes towards EBP.

**Fig 3:**
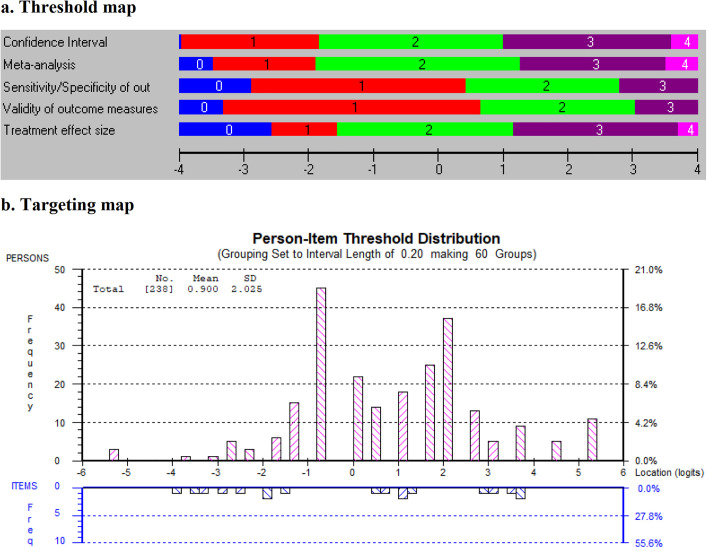
a. Threshold map and b. Targeting map of the “Knowledge about EBP” construct.

Among the initial nine positively worded items, three exhibited disordered thresholds and were rescored by merging the ‘*Strongly disagree’* and *‘Disagree’* categories (items 21, 23, and 25). These same items were subsequently removed due to a misfit with the Rasch model. The final 6-item measure showed no evidence of local item dependency or DIF. It demonstrated good fit to the Rasch model (χ² = 15.08, df = 18, p = 0.66), with no significant dimensionality concerns (% of significant t-tests = 4.98). Fig 3a illustrates the item-threshold gradient, reflecting the hierarchical structure of the items. However, Fig 3b indicates that participants were not well-targeted by the items, with a mean person location of 2.65 (expected 0) and an SD of 1.98 (expected 1). Despite this, the measure exhibited good reliability, with a PSI of 0.84 and a Cronbach’s α of 0.86. Table 8 presents the Rasch analysis results for the positively-worded attitudes towards EBP.

**Table 8:** Rasch analysis results for the Rasch model for the “Positively-worded Attitudes” measure.

a. Fit of data to the Rasch model (n = 238).												
Analysis	N of items	Location, mean (SD)		Fit residuals, mean (SD)		Chi-square interaction		Reliability		Dimensionality	Local item independence	DIF
		Item	Person	Item	Person	χ^2^ (df)	*p*	PSI	Cronbach’s α	% t-tests >5%		
**A1**	9	0 (0.51)	2.17 (1.84)	−0.64 (2.66)	−0.99 (2.03)	48.29 (27)	0.00	0.87	0.91	7.56	None	23 by sex
**A2**	9	0 (0.70)	2.04 (1.87)	−0.30 (3.62)	−0.04 (2.08)	45.77 (27)	0.01	0.87	0.90	5.46	None	23 by sex
**A3**	8	0 (1.10)	2.77 (2.16)	−1.02 (1.68)	−0.93 (1.91)	20.95 (24)	0.64	0.89	0.90	7.98	None	None
**A4**	7	0 (1.08)	2.56 (2.06)	−0.83 (1.26)	−0.85 (1.77)	18.95 (21)	0.59	0.87	0.88	7.14	None	None
**A5**	6	0 (1.04)	2.65 (1.98)	−0.65 (0.83)	−0.75 (1.59)	15.08 (18)	0.66	0.84	0.86	4.98	None	None
***Ideal value***		*0 (1.0)*	*0 (1.0)*	*<1.4*	*<1.4*		*>0.05*	*>0.7*	*>0.7*	*< 5.0*	*<0.3 below average residual*	*None*
**b. Changes applied to the “Positively-worded Attitudes” items.**												
***Instructions:*** *Please indicate your level of agreement with the following statements: 5-point Likert Scale*												
**Item #**	**Description of Item**						**Strongly Disagree**		**Disagree**	**Neutral**	**Agree**	**Strongly agree**
17	New evidence is so important that I make the time in my work schedule.						0		1	2	3	4
18	My practice has changed because of evidence I have found.						0		1	2	3	4
19	Evidence-based practice is fundamental to my professional practice.						0		1	2	3	4
20	I need to increase the use of evidence in my daily practice.						0		1	2	3	4
21	An evidence-based practice approach improves the quality of my practice.						**Deleted because of misfit**					
22	Literature and research findings are useful in my daily practice.						0		1	2	3	4
23	Evidence based practice helps me to make decisions about patients/clients in my practice.						**Deleted because of misfit**					
24	I am willing to use new and different types of clinical interventions (e.g., assessment, treatment) developed by researchers to help my patients/ clients.						0		0	1	2	3
25	I would try a new therapy/intervention even if it were very different from what I am used to doing.						**Deleted because of misfit**					

x: Mean; SD: Standard deviation; χ²: Chi-squared; df: Degrees of freedom; DIF: Differential item functioning; PSI: Person separation index; α: Alpha.

A1: Explored items.

A2: Rescored disordered thresholds of items 21, 24, and 25.

A3: Removed item 25 as it misfits the Rasch model (fit residual = 8.572).

A4: Removed item 23 as it misfits the Rasch model (fit residual = −3.280).

A5: Removed item 21 as it misfits the Rasch model (fit residual = −2.683).

#### Negatively-worded attitudes towards EBP.

**Fig 4:**
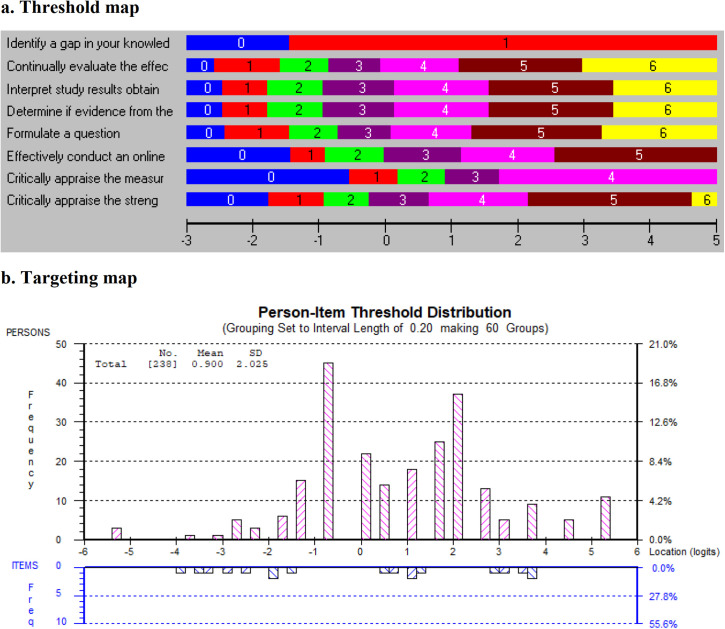
a. Threshold map and b. Targeting map of the “Self-efficacy toward EBP” construct.

All eight negatively worded items were reverse-scored due to their phrasing. None exhibited disordered thresholds, but item 31 was removed due to misfit. The final 7-item measure showed no local item dependency or DIF and demonstrated a good fit to the Rasch model (χ² = 15.23, df = 21, p = 0.81), with no significant dimensionality issues (% of significant t-tests = 4.96). Fig 4a displays the item-threshold gradient, while Fig 4b indicates that participants were well-targeted by the items, with a mean person location of 0.01 (expected 0) and an SD of 1.40 (expected 1). The measure also exhibited good reliability, with a PSI of 0.84 and a Cronbach’s α of 0.86. Table 9 presents the Rasch analysis results for the negatively worded attitudes towards EBP.

**Table 9:** Rasch analysis results for the Rasch model for the “Negatively-worded Attitudes” measure.

a. Fit of data to the Rasch model (n = 238).												
Analysis	N of items	Location, mean (SD)		Fit residuals, mean (SD)		Chi-square interaction		Reliability		Dimensionality	Local item independence	DIF
		Item	Person	Item	Person	χ^2^ (df)	*p*	PSI	Cronbach’s Alpha	% t-tests >5%		
**A1**	8	0 (0.55)	0.07 (1.49)	0.03 (1.31)	−0.54 (1.46)	37.73 (24)	0.04	0.87	0.86	6.30	28 + 29	32 by experience
**A2**	7	0 (0.53)	0.01 (1.40)	0.12 (0.86)	−0.53 (1.40)	15.23 (21)	0.81	0.84	0.83	4.96	None	32 by experience
***Ideal value***		*0 (1.0)*	*0 (1.0)*	*<1.4*	*<1.4*		*>0.05*	*>0.7*	*>0.7*	*< 5.0*	*<0.3 below average residual*	*None*
**b. Changes applied to the “Negatively-worded Attitudes” items**												
***Instructions:*** *Please indicate your level of agreement with the following statements: 5-point Likert Scale*												
Item #	**Description of Item**							**Strongly Disagree**	**Disagree**	**Neutral**	**Agree**	**Strongly agree**
26*	I dislike having my clinical practice questioned.							4	3	2	1	0
27*	I stick to tried and trusted methods in my practice rather than changing to anything new							4	3	2	1	0
28*	Clinical experience is the most reliable way to know what really works							4	3	2	1	0
29*	Clinical experience is more useful than scientific studies when I make decisions about my patients/clients							4	3	2	1	0
30*	Critical appraisal of the literature is not very practical to do in my day-to-day practice							4	3	2	1	0
31*	Seeking relevant evidence from scientific studies is not very practical in the real world							**Deleted because of misfit**				
32*	I know better than academic researchers how to care for my patients/clients							4	3	2	1	0
33*	Research based treatments/interventions are not clinically useful							4	3	2	1	0

x: Mean; SD: Standard deviation; χ²: Chi-squared; df: Degrees of freedom; DIF: Differential item functioning; PSI: Person separation index; α: Alpha.

A1: Explored and reversed all items.

A2: Removed item 31 as it misfits the Rasch model (fit residual = −1.653, χ^2^ = 10.357, p-value = 0.016, F-stat = 5.116, p-value for F-stat = 0.002).

*Item was reversed because of negative meaning.

### Resources

**Fig 5:**
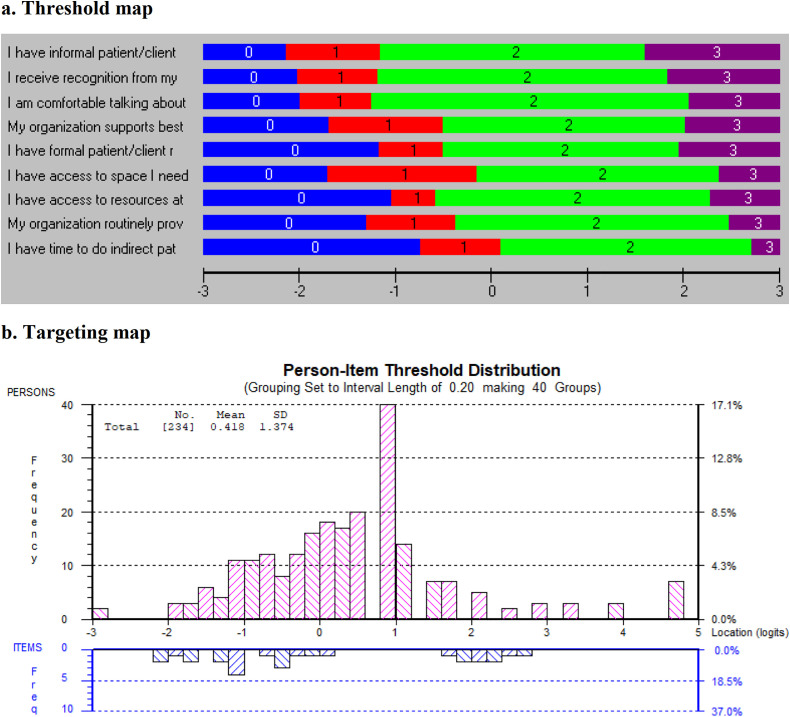
a. Threshold map and b. Targeting map of the “Resources” construct.

Item 65 was reverse-scored due to its negative phrasing (Table 10). Initially, all items in the resources construct exhibited disordered thresholds, which were addressed by merging the ‘Strongly disagree’ and ‘Disagree’ response categories. Seven items (55, 57, 59, 64, 65, 67, and 68) were subsequently removed due to a misfit with the Rasch model. The final 8-item measure showed no evidence of local item dependency or DIF. The final model demonstrated a good fit to the Rasch model (χ² = 26.60, df = 27, p = 0.49), with no significant dimensionality concerns (% of significant t-tests = 4.89). Fig 5a illustrates the gradient across item thresholds, reflecting the hierarchical structure of the items. Fig 5b indicates that participants were reasonably well-targeted by the items, with a mean person location of 0.42 (expected 0) and an SD of 1.37 (expected 1).

**Table 10:** Rasch analysis results for the Rasch model for the “Resources” measure.

a. Fit of data to the Rasch model (n = 238).												
Analysis	N of items	Location, mean (SD)		Fit residuals, mean (SD)		Chi-square interaction		Reliability		Dimensionality	Local item independence	DIF
		Item	Person	Item	Person	χ^2^ (df)	*p*	PSI	Cronbach’s α	% t-tests >5%		
**A1**	15	0 (0.82)	0.99 (0.97)	0.26 (3.13)	−0.58 (2.03)	190.25 (45)	0.000	0.86	0.86	14.53	54 + 55, 60 + 61, 60 + 62, 62 + 63, 56 + 66, 66 + 67, 67 + 68	None
**A2**	15	0.0 (0.57)	0.24 (1.08)	0.13 (2.83)	−0.55 (2.02)	156.97 (45)	0.000	0.87	0.87	13.57	54 + 55, 60 + 61, 60 + 62, 62 + 63, 56 + 66, 66 + 67, 67 + 68	None
**A3**	14	0.0 (0.55)	0.35 (1.26)	−0.03 (2.09)	−0.81 (2.40)	104.74 (42)	0.000	0.88	0.88	8.33	54 + 55, 60 + 61, 66 + 67, 67 + 68	None
**A4**	13	0.0 (0.56)	0.33 (1.35)	−0.08 (1.65)	−0.79 (2.29)	51.72 (39)	0.084	0.88	0.87	7.44	54 + 55, 60 + 61, 66 + 67, 67 + 68	None
**A5**	12	0.0 (0.44)	0.47 (1.47)	−0.18 (0.93)	−.87 (2.27)	50.21 (36)	0.058	0.88	0.89	8.84	54 + 55, 60 + 61, 66 + 67, 67 + 68	None
**A6**	11	0.0 (0.45)	0.43 (1.44)	−0.15 (0.86)	−0.85 (2.18)	40.28 (33)	0.179	0.87	0.88	9.72	54 + 55, 60 + 61	None
**A7**	10	0.0 (0.43)	0.38 (1.43)	−0.19 (0.95)	−0.83 (2.10)	34.64 (30)	0.256	0.86	0.86	5.2	None	None
**A8**	9	0.0 (0.41)	0.42 (1.37)	−0.24 (1.07)	−0.81	26.60 (27)	0.486	0.84	0.84	4.89	None	None
***Ideal value***		*0 (1.0)*	*0 (1.0)*	*<1.4*	*<1.4*		*>0.05*	*>0.7*		*< 5.0*	*<0.3 below average residual*	*None*
**b. Changes applied to the “Resources” items.**												
***Instructions:*** *Please indicate your level of agreement with the following statements with respect to your organization or workplace setting: 5-point Likert*												
**Item #**	**Description of Item**						**Strongly Disagree**		**Disagree**	**Neutral**	**Agree**	**Strongly agree**
54	I am comfortable talking about patient/client care issues with those in charge in my organization						0		1	2	3	
55	I receive recognition from my manager(s)/supervisor(s) about my work						**Deleted because of misfit**					
56	I have control over *how* I do my work						0		1	2	3	
57	My organization emphasizes productivity						**Deleted because of misfit**					
58	My organization supports best practice						0		1	2	3	
59	I have opportunities for educational activities in my organization						**Deleted because of misfit**					
60	I have formal patient/client related discussions with peers or colleagues (e.g., continuing education, patient rounds, team meetings) in my organization						0		1	2	3	
61	I have informal patient/client related discussions with peers or colleagues (e.g., other health care providers, informal bedside teaching) in my organization						0		1	2	3	
62	My organization routinely provides information/ feedback on my practice (e.g., audits, performance reviews)						0		1	2	3	
63	I have access to resources at my workplace to help deliver quality care for my patients/clients (e.g., databases, libraries, equipment)						0		1	2	3	
64	All positions in my profession are currently filled at my workplace						**Deleted because of misfit**					
65*	There is a high turnover rate of clinicians in my profession at my workplace						**Deleted because of misfit**					
66	I have access to space I need to do my job well at my workplace						0		1	2	3	
67	There is appropriate space to provide quality care						**Deleted because of misfit**					
68	I have time to do indirect patient activities (e.g., talk about a plan of care, look up something in a journal, get involved in new initiatives at work) in my practice						**Deleted because of misfit**					

x: Mean; SD: Standard deviation; χ²: Chi-squared; df: Degrees of freedom; DIF: Differential item functioning; PSI: Person separation index; α: Alpha.

A1: Explore items and reverse item 65.

A2: Rescore disordered thresholds for all items.

A3: Remove item 65 as it misfits the Rasch model (fit residual = 7.782).

A4: Remove item 57 as it misfits the Rasch model (fit residual = 5.154).

A5: Remove item 64 as it misfits the Rasch model (fit residual = 4.6910).

A6: Remove item 59 as it misfits the Rasch model (fit residual = −1.415, χ^2^ = 9.169, p-value = 0.0271, F-stat = 4.085, p-value for F-stat = 0.0075).

A7: Remove item 55 as it misfits the Rasch model (fit residual = −0.623, χ^2^ = 9.209, p-value = 0.0266, F-stat = 3.651, p-value for F-stat = 0.0134).

A8: Remove item 67 as it misfits the Rasch model (fit residual = −1.369, χ^2^ = 6.006, p-value = 0.1114, F-stat = 2.718, p-value for F-stat = 0.0454).

A9: Remove item 68 as it misfits the Rasch model (fit residual = −1.73, χ^2^ = 4.907, p-value = 0.086003, F-stat = 3.2109, p-value for F-stat = 0.04263).

*Item was reversed because of negative meaning.

## Discussion

This study aimed to cross-culturally adapt and validate an EBP measure for healthcare providers in Hong Kong, ensuring its linguistic, cultural, and psychometric appropriateness. The findings demonstrate that the adapted EBP measure is a robust tool for evaluating EBP-related constructs among these providers, with strong reliability and validity across its six constructs.

Minimal modifications were required to simplify language and align with local terminology, reflecting the shared professional language of English in Hong Kong’s healthcare system. High relevance and comprehensibility ratings from cognitive debriefing further support the measure’s cultural appropriateness.

The use of EBP and EBP activities constructs, which follow a formative model, requires no substantive changes beyond linguistic adjustments. These constructs provide valuable insights into the frequency and types of EBP activities undertaken by healthcare providers, offering a practical framework for evaluating EBP integration into clinical practice. Psychometric validation through Rasch analysis revealed strong construct validity and reliability for most constructs of the reflexive model.

The knowledge construct underwent significant refinement, with six misfitting items removed. The final 5-item measure showed excellent reliability and unidimensionality, with a clear hierarchy of item difficulty. This suggests that while participants were generally familiar with basic EBP concepts, more advanced topics, such as treatment effect size, posed greater challenges. However, it is important to consider that healthcare providers may not need to master these intricate details to engage in EBP effectively [36]. Instead, focusing on fundamental competencies that support the practical application of EBP in daily clinical settings may be more beneficial. These findings highlight potential gaps in EBP knowledge among Hong Kong healthcare providers, which could inform future educational interventions by emphasizing essential skills that enhance clinical practice.

Self-efficacy and resources also demonstrated strong psychometric properties. The rescoring of disordered thresholds and removal of misfitting items improved model fit, ensuring that the measures accurately reflect participants’ confidence in applying EBP and their access to EBP-related resources. The reliability scores for these constructs further validate their utility in assessing EBP implementation.

However, the attitudes construct was found to be multidimensional, requiring its division into positively and negatively worded subscales. This was not necessary when validating the measure among physiotherapy and occupational therapy graduates [15] or physiotherapy students [17]. Despite these findings, both subscales demonstrated good fit and reliability, supporting their use as distinct measures.

This finding suggests that attitudes toward EBP may not be adequately captured as a single unidimensional construct in this population. Rather, the positively and negatively worded items appear to reflect two related but distinct dimensions of attitudes. In practical terms, this supports interpreting the attitudes domain as two separate subscales rather than a single overall score when used among Hong Kong healthcare providers. At the same time, this result also indicates that further refinement of the attitudes construct may be warranted in future research to determine whether a more coherent unidimensional scale can be developed for this population.

This division likely reflects differences in professional experience between practicing clinicians and students or recent graduates. As Thomas et al. [37] discuss, attitudes toward EBP are complex and shaped by factors such as social desirability and exposure to real-world constraints. While students’ perspectives are often shaped by academic training and emphasize the idealized benefits of EBP [38,39], practicing clinicians navigate barriers like time constraints, resource limitations, and organizational challenges [40]. These challenges may lead to more nuanced or critical attitudes, particularly in response to negatively worded items [37]. Cognitive load differences may also impact how clinicians interpret these items, potentially introducing measurement error [41,42]. Negatively worded items were intentionally retained from the original measure in part to reduce acquiescence bias; however, such items may also introduce wording-related method effects. Because items were presented in construct-based sections rather than randomized order, some response-style bias may still have been present. Given these factors, treating positively- and negatively-worded attitudes as distinct constructs improves measurement accuracy in heterogeneous populations [43]. This approach provides a more detailed understanding of how attitudes influence EBP implementation, allowing for targeted educational and support strategies.

The multidimensionality of the attitudes construct is consistent with previous research that used different attitude scales, demonstrating that combining positively and negatively worded items can introduce artificial factors and undermine unidimensionality [44–47]. Weijters and Baumgartner (2012) [48] found that combining positively and negatively worded items in the same scale can result in multidimensionality. To improve validity and interpretability, it is recommended that such items be separated [44]. The literature supports our decision to divide the attitudes construct into two subscales in order to accurately represent a single underlying dimension.

These findings have practical implications for future use of the measure. The adapted instrument can be used to assess key EBP-related constructs among healthcare providers in Hong Kong, but the attitudes domain may be more appropriately interpreted as two separate subscales rather than as a single overall score. This distinction is important for both researchers and educators, as it allows a more nuanced understanding of clinicians’ endorsement of EBP and their perceived reservations or barriers. Future research should examine whether refinement of item wording or construct structure can improve coherence while preserving conceptual breadth.

The Rasch analysis revealed a notable lack of middle-range items across most EBP constructs, except for negatively worded attitudes toward EBP. This gap suggests that the measure may not fully capture the spectrum of EBP competencies, particularly for individuals with moderate engagement or understanding. The absence of middle-range items could result from a skewed item pool or contextual differences in Hong Kong’s healthcare system that shape how EBP is practiced and perceived [49]. While this issue was not observed in the original [15] or student [17] versions of the measure, it highlights the need for further item development. Future research should focus on expanding the item pool, potentially through qualitative methods such as interviews or focus groups, to better reflect the diverse experiences of healthcare providers.

Recent studies emphasize dynamic frameworks that account for evolving clinical environments, shifting patient demographics, and the increasing complexity of healthcare systems in assessing attitudes toward EBP [50,51]. While quantitative measures provide valuable standardized assessments, there is growing recognition that they may not fully capture the contextual and experiential factors influencing EBP adoption [52]. Some scholars advocate for mixed-method approaches, arguing that qualitative data—such as interviews and focus groups—can reveal deeper insights into barriers, facilitators, and real-world applications of EBP that quantitative scales alone might overlook [37,53]. The field may need to move toward a more integrated approach, combining multiple tools and data sources to gain a fuller and more nuanced understanding of EBP [54]. By leveraging both qualitative and quantitative methods, we can develop more comprehensive measurement strategies that better reflect the complexities of clinical practice and support meaningful advancements in EBP implementation.

Our study employed self-reported data to evaluate participants’ EBP knowledge, skills, and activities. While self-reported measures are practical and offer valuable insights, they inherently depend on participants’ perceptions, which may not always perfectly align with their actual abilities. This is a common consideration in survey-based research. In addition, self-reported Rasch ordinal data may be affected by potential biases, such as social desirability bias due to overreporting positive behaviors [55–57], recall bias [58], or certain response styles, such as participants of certain cultures consistently choosing extreme options [59]. Such biases can influence the normal distribution of responses and may affect the accuracy of Rasch model estimates, leading to measurement error [60].

Future studies could improve the evaluation of EBP education by integrating self-reported data with performance-based assessments, particularly for tasks that require specific skills, such as developing research keywords and conducting literature searches. However, within the scope and constraints of our study, self-reported data remains a valuable tool for capturing participants’ self-assessed competencies and experiences.

## Strengths and limitations

This study had several strengths. First, its rigorous cross-cultural adaptation and comprehensive psychometric validation ensure the measure’s cultural appropriateness and reliability for Hong Kong healthcare providers. The use of Rasch analysis confirmed the measure’s unidimensionality and construct validity while addressing misfitting items and local dependencies. Second, its methodological rigor, transparency, and adherence to the STROBE checklist enhance the credibility and reproducibility of the findings [16,18]. Third, strong internal consistency reliability, with Cronbach’s α and PSI values exceeding 0.7 for all constructs, underscores the measure’s robustness [33,34]. Fourth, by capturing both individual and organizational factors, the measure provides a holistic assessment of EBP competencies, supporting continuous improvement in healthcare delivery and ultimately enhancing patient outcomes.

However, several limitations that warrant consideration. First, the sample size, while sufficient for Rasch analysis, may not fully represent the diversity of healthcare professions in Hong Kong, potentially limiting the generalizability of the findings. Second, the study’s cross-sectional design, focused on cross-cultural validation and psychometric assessment, precludes the evaluation of test-retest reliability, which is essential for assessing the stability of the measure over time. Third, the exclusion of certain healthcare professions or those with limited English proficiency may further restrict the measure’s applicability across all provider groups such as traditional Chinese medicine practitioners. Fourth, while the measure was adapted for Hong Kong, its use in other cultural or linguistic contexts would require additional validation to ensure its relevance and accuracy. Fifth, some items removed due to misfit in the Rasch analysis might be important to EBP, although similar concepts may be covered by other items in the measure. While their deletion was necessary to maintain the psychometric integrity of the scale, we acknowledge that these changes may have resulted in a narrower representation of certain EBP dimensions. Sixth, the scoring of the formative constructs. For Use of EBP, the dichotomous cutoff of “at least once” was retained from the original instrument, while for EBP Activities, weighted response options were used to approximate the number of activity occasions per month. Although these approaches are conceptually consistent with the original formative model, they may still reduce granularity or affect score distributions. We did not conduct sensitivity analyses using alternative thresholds or scoring structures in the present sample, and this should be explored in future studies. Future research could explore alternative item formulations or supplementary approaches to ensure comprehensive coverage of all relevant aspects of EBP.

## Conclusions

This study successfully adapted and validated an EBP measure for use among Hong Kong healthcare providers. The measure’s strong psychometric properties and cultural relevance make it a valuable tool for assessing and enhancing EBP competencies in this population. By identifying gaps in knowledge, self-efficacy, and resource availability, this measure can inform targeted interventions to promote evidence-based care, ultimately improving patient outcomes and healthcare quality in Hong Kong.

## Supporting information

S1 File Changes to the original questionnaires and the pilot study results. (DOCX)

S1 Appendix List of item hierarchy of each construct. (PDF)
