# Supplementary material for: Cross-cultural adaptation of evidence-based practice measure among Hong Kong healthcare providers
Source: PLoS One. 2026 Jun 26;21(6):e0351754. doi: 10.1371/journal.pone.0351754 (PMC13308815; doi:10.1371/journal.pone.0351754)
Supplement: S1 File — (DOCX) [file pone.0351754.s001.docx]

**S1 File: Changes to the original questionnaires and the pilot study results**

**Current self-reported use of Evidence-based practice**

| **Changes applied to the items after forward and backward translation** | | | | | | | **Test of the pre-final version level of (n = 36)** | | | |
| --- | --- | --- | --- | --- | --- | --- | --- | --- | --- | --- |
| ***Instructions:*** *~~For each of the following activities, how often have you done the following in the~~* ***~~past 6 months~~ (****In the* ***past 6 months****, how often have you****)****? 5-point Scale* | | | | | | | ***Relevance*** | | ***Comprehension*** | |
| **Item #** | **Description of Item:** | **Never** | **1 to 2 times** | **Almost every month** | **2 to 10 times a month** | **More than 10 times a month** | **Mean** | **SD** | **Mean** | **SD** |
| 1 | ~~Identify~~ (Identified) a gap in your knowledge related to a patient or client situation (e.g. history, assessment, treatment)? |  |  |  |  |  | 3.8 | 1.1 | 3.8 | 1.2 |
| 2 | ~~Formulate~~ (Formulated) a question to guide a literature search based on a gap in your knowledge? |  |  |  |  |  | 3.6 | 1.3 | 4.1 | 1.1 |
| 3 | Effectively ~~conduct~~ (conducted) an online literature search to address the question? |  |  |  |  |  | 3.7 | 1.3 | 4.0 | 1.1 |
| 4 | Critically ~~appraise~~ (appraised) the strengths and weaknesses of ~~study~~ (research) methods (e.g. appropriateness of study design, recruitment, data collection and analysis)? |  |  |  |  |  | 3.3 | 1.3 | 3.9 | 1.1 |
| 5 | Critically ~~appraise~~ (appraised) the measurement properties (e.g. reliability and validity, sensitivity and specificity) of standardized tests or assessment tools you are considering using in your practice? |  |  |  |  |  | 3.3 | 1.3 | 3.9 | 1.1 |
| 6 | ~~Interpret~~ (Interpreted) study results ~~obtained using~~ (with the use of) statistical tests and procedures (e.g. t-tests, logistic regression?) |  |  |  |  |  | 2.9 | 1.4 | 3.8 | 1.3 |
| 7 | ~~Determine~~ (Determined) if evidence from the research literature applies to your patient's/client's situation? |  |  |  |  |  | 4.0 | 1.0 | 4.2 | 1.1 |
| 8 | ~~Decide~~ (Determined) on an appropriate course of action based on integrating the research evidence, clinical judgment and patient or client preferences? |  |  |  |  |  | 3.9 | 1.1 | 4.1 | 0.9 |
| 9 | Continually ~~evaluate~~ (evaluated) the effect of your course of action on your patient's/client's outcomes? |  |  |  |  |  | 4.2 | 0.9 | 4.3 | 0.9 |

**EBP activities**

| **Changes applied to the items after forward and backward translation** | | | | | | | **Test of the pre-final version level of (n = 36)** | | | |
| --- | --- | --- | --- | --- | --- | --- | --- | --- | --- | --- |
| ***Instructions:*** *In the* ***past month*** *how often have you?: 5-point Scale* | | | | | | | ***Relevance*** | | ***Comprehension*** | |
| **Item #** | **Description of Item** | **Never** | **Monthly or less** | **Bi-weekly** | **Weekly** | **Daily** | **Mean** | **SD** | **Mean** | **SD** |
| 10 | Integrated research evidence with your expertise |  |  |  |  |  | 3.8 | 1.2 | 4.1 | 1.1 |
| 11 | ~~Informally (e.g. outside of formal team or family meetings) shared and discussed literature/research findings with colleagues at work~~  Informally (e.g. hallway chatting) shared and discussed literature/research findings with colleagues at work |  |  |  |  |  | 4.1 | 1.1 | 4.3 | 1.0 |
| 12 | ~~Formally (e.g. during team or family meetings) shared and discussed literature/research findings with colleagues at work~~  Formally (e.g. during team meetings) shared and discussed literature/research findings with colleagues at work |  |  |  |  |  | 3.8 | 1.2 | 4.0 | 1.2 |
| 13 | Shared and discussed literature/research findings with patients/clients |  |  |  |  |  | 3.8 | 1.2 | 4.1 | 1.1 |
| 14 | Read published research reports |  |  |  |  |  | 3.9 | 1.1 | 4.3 | 0.9 |
| 15 | ~~Made~~ (Spent) time to read research |  |  |  |  |  | 3.9 | 1.1 | 4.3 | 0.8 |
| 16 | Attended in-services/workshops/courses in your organization? |  |  |  |  |  | 3.9 | 1.3 | 4.2 | 1.2 |

**Attitudes towards EBP**

| **Changes applied to the items after forward and backward translation** | | | | | | | **Test of the pre-final version level of (n = 36)** | | | |
| --- | --- | --- | --- | --- | --- | --- | --- | --- | --- | --- |
| ***Instructions****: Please indicate your level of agreement with the following statements: 5-point Likert Scale* | | | | | | | ***Relevance*** | | ***Comprehension*** | |
| **Item #** | **Description of Item** | **Strongly Disagree** | **Disagree** | **Neutral** | **Agree** | **Strongly agree** | **Mean** | **SD** | **Mean** | **SD** |
| 17 | New evidence is so important that I make the time in my work schedule. |  |  |  |  |  | 4.0 | 1.2 | 4.3 | 1.1 |
| 18 | My practice has changed because of evidence I have found. |  |  |  |  |  | 4.1 | 1.3 | 4.5 | 1.1 |
| 19 | Evidence-based practice is fundamental to my professional practice. |  |  |  |  |  | 4.3 | 1.2 | 4.5 | 1.0 |
| 20 | I need to increase the use of evidence in my daily practice. |  |  |  |  |  | 4.1 | 1.2 | 4.5 | 1.3 |
| 21 | An evidence-based practice approach improves the quality of my practice. |  |  |  |  |  | 4.2 | 1.3 | 4.5 | 1.0 |
| 22 | Literature and research findings are useful in my daily practice. |  |  |  |  |  | 4.1 | 1.2 | 4.4 | 1.0 |
| 23 | Evidence based practice helps me to make decisions about patients/clients in my practice. |  |  |  |  |  | 4.2 | 1.2 | 4.5 | 1.0 |
| 24 | I am willing to use new and different types of clinical interventions (e.g. assessment, treatment) developed by researchers to help my patients/ clients. |  |  |  |  |  | 4.2 | 1.2 | 4.5 | 1.1 |
| 25 | I would try a new therapy/intervention even if it were very different from what I am used to doing. |  |  |  |  |  | 4.1 | 1.1 | 4.3 | 1.1 |
| 26 | I resent (dislike) having my clinical practice questioned. |  |  |  |  |  | 4.0 | 1.2 | 4.2 | 1.3 |
| 27 | I stick to tried and trusted methods in my practice rather than changing to anything new |  |  |  |  |  | 4.0 | 1.2 | 4.3 | 1.1 |
| 28 | Clinical experience is the most reliable way to know what really works |  |  |  |  |  | 4.1 | 1.1 | 4.3 | 1.1 |
| 29 | Clinical experience is more useful than scientific studies when I make decisions about my patients/clients |  |  |  |  |  | 4.1 | 1.2 | 4.4 | 1.1 |
| 30 | Critical appraisal of the literature is not very practical to do in my day-to-day practice |  |  |  |  |  | 3.9 | 1.2 | 4.2 | 1.2 |
| 31 | Seeking relevant evidence from scientific studies is not very practical in the real world |  |  |  |  |  | 3.9 | 1.3 | 4.3 | 1.2 |
| 32 | I know better than academic researchers how to care for my patients/clients |  |  |  |  |  | 3.9 | 1.3 | 4.3 | 1.1 |
| 33 | Research based treatments/interventions are not clinically useful |  |  |  |  |  | 3.9 | 1.3 | 4.3 | 1.2 |

**Knowledge of EBP**

| **Changes applied to the items after forward and backward translation** | | | | | | | **Test of the pre-final version level of (n = 36)** | | | |
| --- | --- | --- | --- | --- | --- | --- | --- | --- | --- | --- |
| ***Instructions:*** *Please rate your understanding of the following terms: 5-point Likert Scale* | | | | | | | ***Relevance*** | | ***Comprehension*** | |
| **Item #** | **Description of Item** | **Never heard the term** | **Have heard it but don't understand** | **Have some understanding** | **Understand quite well** | **Understand and could explain to others** | **Mean** | **SD** | **Mean** | **SD** |
| 34 | Reliability of outcome measures |  |  |  |  |  | 4.1 | 1.1 | 4.3 | 1.2 |
| 35 | Validity of outcome measures |  |  |  |  |  | 4.1 | 1.2 | 4.3 | 1.2 |
| 36 | Sensitivity/Specificity of outcome measures |  |  |  |  |  | 4.0 | 1.3 | 4.3 | 1.0 |
| 37 | Meta-analysis |  |  |  |  |  | 3.7 | 1.4 | 4.2 | 1.3 |
| 38 | Confidence Interval |  |  |  |  |  | 3.7 | 1.4 | 4.1 | 1.3 |
| 39 | Systematic Review |  |  |  |  |  | 4.0 | 1.2 | 4.3 | 1.1 |
| 40 | Number needed to treat |  |  |  |  |  | 3.6 | 1.3 | 4.0 | 1.3 |
| 41 | Statistical significance |  |  |  |  |  | 3.9 | 1.2 | 4.2 | 1.1 |
| 42 | Minimally important change (MIC) |  |  |  |  |  | 3.5 | 1.4 | 4.0 | 1.3 |
| 43 | Treatment effect size |  |  |  |  |  | 3.9 | 1.2 | 4.0 | 1.3 |
| 44 | Randomized controlled trial (RCT) |  |  |  |  |  | 4.0 | 1.2 | 4.3 | 1.1 |

**Confidence towards EBP**

| **Changes applied to the items after forward and backward translation** | | | | | | | | | | | | | **Test of the pre-final version level of (n = 36)** | | | |
| --- | --- | --- | --- | --- | --- | --- | --- | --- | --- | --- | --- | --- | --- | --- | --- | --- |
| ***Instructions:*** *Please indicate how confident you are in your current level of ability by choosing the corresponding number on the following rating scale: 11-point Continuous Scale* | | | | | | | | | | | | | ***Relevance*** | | ***Comprehension*** | |
| **Item #** | **Description of Item** | **0% (No confidence)** | **10%** | **20%** | **30%** | **40%** | **50%** | **60%** | **70%** | **80%** | **90%** | **100% (Completely confident)** | **Mean** | **SD** | **Mean** | **SD** |
| 45 | Identify a gap in your knowledge related to a patient or client situation (e.g. history, assessment, treatment)? |  |  |  |  |  |  |  |  |  |  |  | 4.1 | 1.4 | 4.2 | 1.4 |
| 46 | Formulate a question to guide a literature search based on a gap in your knowledge? |  |  |  |  |  |  |  |  |  |  |  | 4.0 | 1.4 | 4.2 | 1.5 |
| 47 | Effectively conduct an online literature search to address the question? |  |  |  |  |  |  |  |  |  |  |  | 3.9 | 1.4 | 4.3 | 1.5 |
| 48 | Critically appraise the strengths and weaknesses of study methods (e.g. appropriateness of study design, recruitment, data collection and analysis)? |  |  |  |  |  |  |  |  |  |  |  | 3.9 | 1.5 | 4.3 | 1.5 |
| 49 | Critically appraise the measurement properties (e.g. reliability and validity, sensitivity and specificity) of standardized tests or assessment tools (that) you are considering using in your practice? |  |  |  |  |  |  |  |  |  |  |  | 3.8 | 1.5 | 4.3 | 1.5 |
| 50 | Interpret study results obtained using statistical tests and procedures (e.g. t-tests, logistic regression?) |  |  |  |  |  |  |  |  |  |  |  | 3.7 | 1.6 | 4.2 | 1.5 |
| 51 | Determine if evidence from the research literature applies to your patient's/client's situation? |  |  |  |  |  |  |  |  |  |  |  | 4.1 | 1.4 | 4.3 | 1.5 |
| 52 | Decide on an appropriate course of action based on integrating the research evidence, clinical judgment and patient or client preferences? |  |  |  |  |  |  |  |  |  |  |  | 4.0 | 1.4 | 4.3 | 1.5 |
| 53 | Continually evaluate the effect of your course of action on your patient's/client's outcomes? |  |  |  |  |  |  |  |  |  |  |  | 4.0 | 1.5 | 4.2 | 1.5 |

**Resources**

| **Changes applied to the items after forward and backward translation** | | | | | | | **Test of the pre-final version level of (n = 36)** | | | |
| --- | --- | --- | --- | --- | --- | --- | --- | --- | --- | --- |
| ***Instructions****: Please indicate your level of agreement with the following statements with respect to your organization or workplace setting: 5-point Likert* | | | | | | | ***Relevance*** | | ***Comprehension*** | |
| **Item #** | **Description of Item** | **Strongly Disagree** | **Disagree** | **Neutral** | **Agree** | **Strongly agree** | **Mean** | **SD** | **Mean** | **SD** |
| 54 | I am comfortable talking about patient/client care issues with those in charge ~~at the~~ (in my) organization |  |  |  |  |  | 4.2 | 1.5 | 4.4 | 1.4 |
| 55 | I receive recognition from my manager(s)/supervisor(s) about my work |  |  |  |  |  | 4.0 | 1.5 | 4.4 | 1.4 |
| 56 | I have control over *how* I do my work |  |  |  |  |  | 4.0 | 1.5 | 4.5 | 1.4 |
| 57 | My organization emphasizes productivity |  |  |  |  |  | 4.1 | 1.5 | 4.4 | 1.4 |
| 58 | My organization supports best practice |  |  |  |  |  | 4.3 | 1.4 | 4.5 | 1.4 |
| 59 | I have opportunities for educational activities in my organization |  |  |  |  |  | 4.2 | 1.5 | 4.4 | 1.5 |
| 60 | I have formal patient/client related discussions with peers or colleagues (e.g. continuing education, patient rounds, team meetings) in my organization |  |  |  |  |  | 4.1 | 1.5 | 4.5 | 1.4 |
| 61 | I have informal patient/client related discussions with peers or colleagues (e.g. other health care providers, informal bedside teaching) in my organization |  |  |  |  |  | 4.1 | 1.4 | 4.4 | 1.4 |
| 62 | My organization routinely provides information/ feedback on my practice (e.g. audits, performance reviews) |  |  |  |  |  | 4.3 | 1.4 | 4.4 | 1.4 |
| 63 | I have access to resources at my workplace to help deliver quality care for my patients/clients (e.g. databases, libraries, equipment) |  |  |  |  |  | 4.1 | 1.5 | 4.5 | 1.4 |
| 64 | All ~~OT/PT~~ positions ~~at my workplace~~ in my profession are currently filled at my workplace |  |  |  |  |  | 3.8 | 1.6 | 4.1 | 1.5 |
| 65* | There is a high ~~OT/PT clinician staff~~ turnover rate of clinicians in my profession at my workplace |  |  |  |  |  | 3.5 | 1.6 | 4.0 | 1.6 |
| 66 | I have access to space I need to do my job well at my workplace |  |  |  |  |  | 3.9 | 1.5 | 4.1 | 1.5 |
| 67 | There is appropriate space to provide quality care |  |  |  |  |  | 4.2 | 1.4 | 4.4 | 1.4 |
| 69 | I have time to do indirect patient activities (e.g. talk about a plan of care, look up something in a journal, get involved in new initiatives at work) in my practice |  |  |  |  |  | 4.0 | 1.6 | 4.2 | 1.6 |
| *Item was reversed because of negative meaning. | | | | | | | | | | |
