# Supplementary material for: Cross-cultural adaptation of evidence-based practice measure among Hong Kong healthcare providers
Source: PLoS One. 2026 Jun 26;21(6):e0351754. doi: 10.1371/journal.pone.0351754 (PMC13308815; doi:10.1371/journal.pone.0351754)
Supplement: S1 Appendix — (PDF) [file pone.0351754.s002.pdf]

S1 Appendix. List of item hierarchy of each construct

Knowledge about EBP

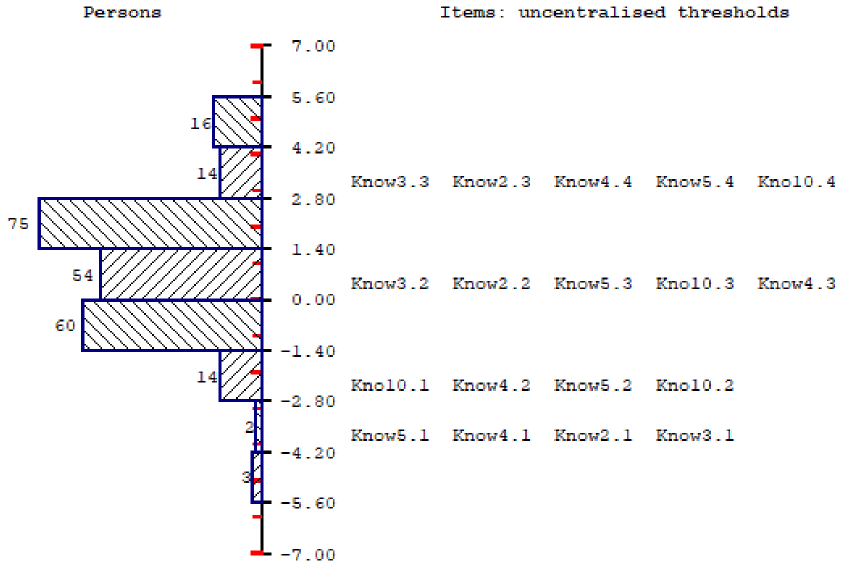

Self-efficacy

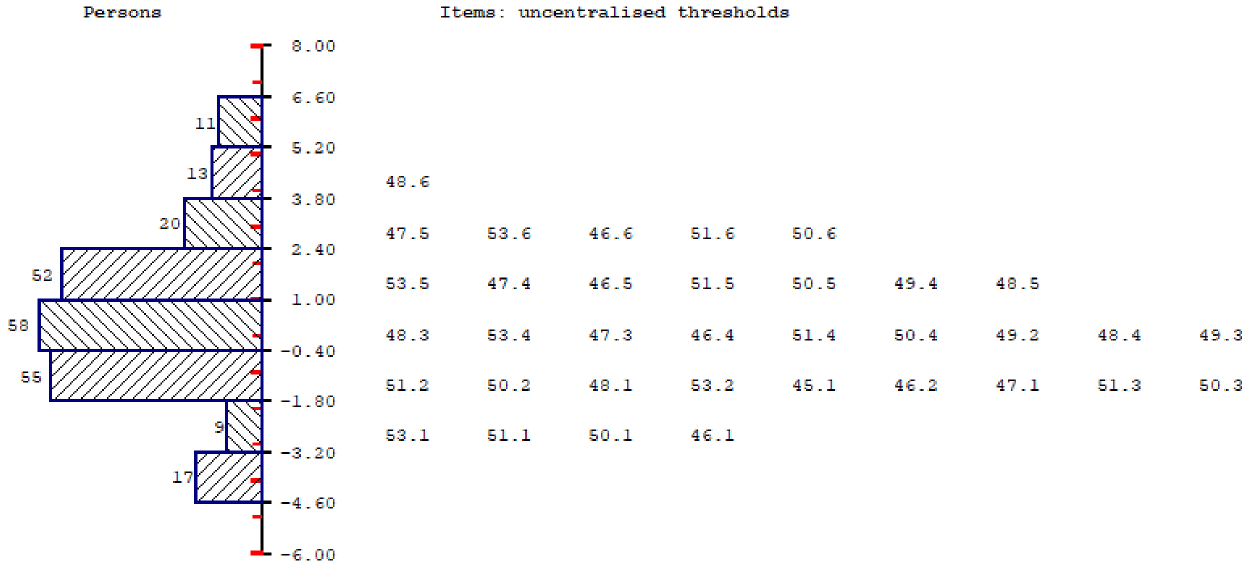

Attitudes towards EBP – Positively worded

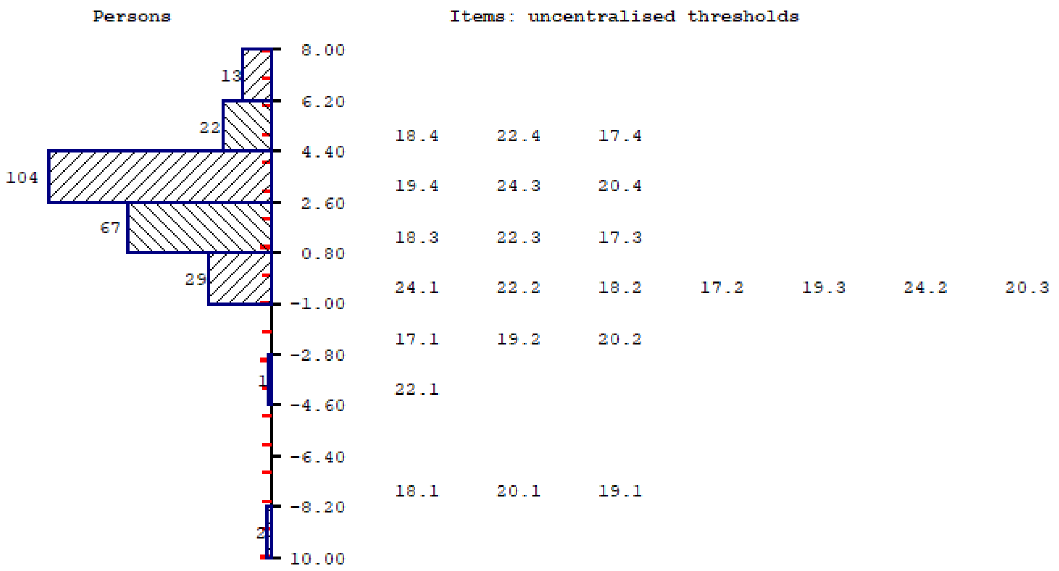

Attitudes towards EBP – Negatively worded

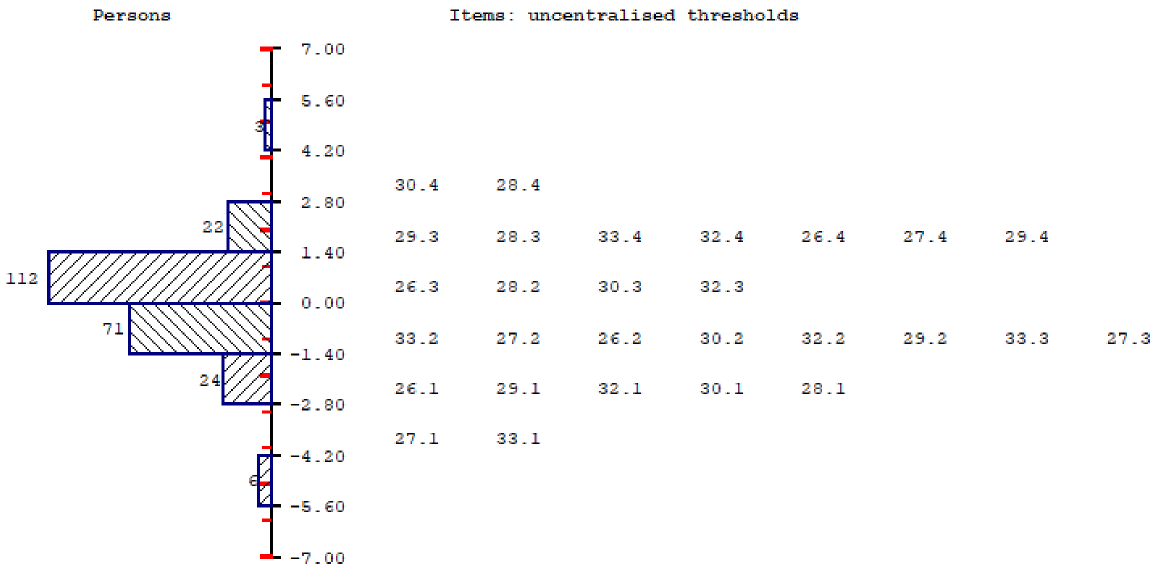

EBP Resources

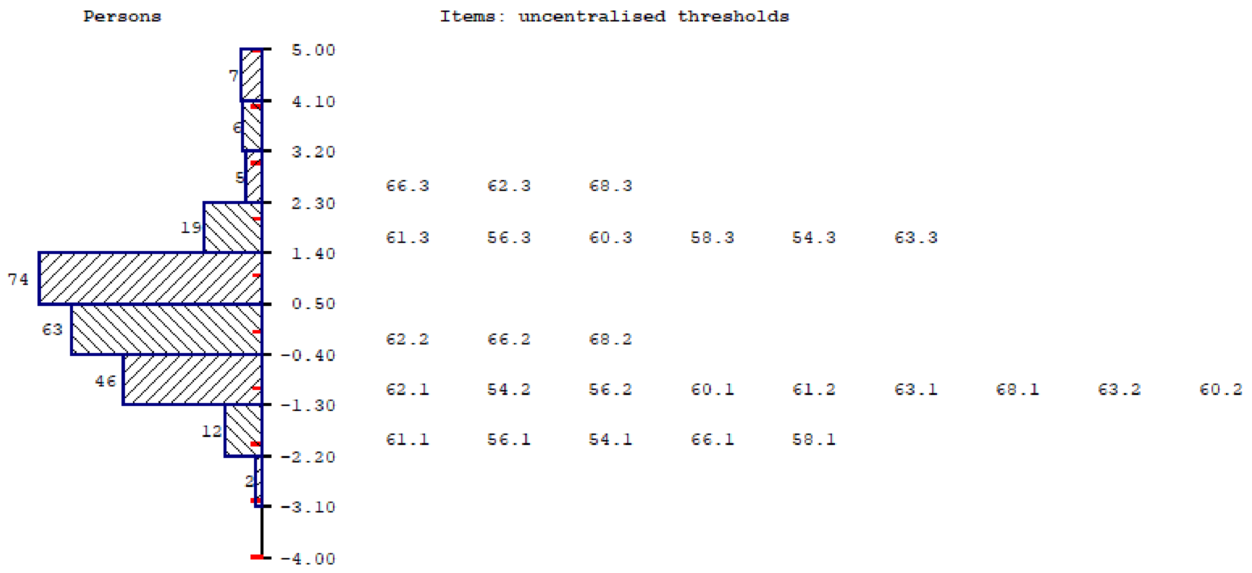
Persons
Items: uncentralised thresholds
5.00
4.10
3.20
2.30
1.40
0.50
-0.40
-1.30
-2.20
-3.10
-4.00
7
6
5
19
74
63
46
12
2
66.3 62.3 68.3
61.3 56.3 60.3 58.3 54.3 63.3
62.2 66.2 68.2
62.1 54.2 56.2 60.1 61.2 63.1 68.1 63.2 60.2
61.1 56.1 54.1 66.1 58.1
